# Acoustic predation in a sailfish-flying fish cloak

**DOI:** 10.1038/s41598-023-40986-w

**Published:** 2023-08-24

**Authors:** Promode R. Bandyopadhyay

**Affiliations:** 1 Naval Undersea Warfare Center, Newport, RI 02841 USA; 2 Present Address: Middletown, RI 02842 USA

**Keywords:** Biophysics, Engineering, Mechanical engineering

## Abstract

When a sailfish circles to corral a school of flying fish in a vortex near the ocean surface, a tiny patch of arced surface waves confined to oppositely placed 70° sectors appears dispersing coherently, but why? It is modeled that, when the fish motions stop suddenly, the corralled school compacts, the tail shed propulsion vortices touch, break and radiate the pressure released from the centrifugal vortex rotation creating an acoustic monopole. The surface-wave patch is a section of the sphere of radiation. The oppositely placed curved bodies of the sailfish and the flying fish act as concave acoustic mirrors about the monopole creating a reverberating bell-shaped cloak in between which vibrates the ear bones and bladders of the flying fish disorienting them. A cup of water firmly struck on a table induces a similar vibration of a purely radial mode. The sailfish circles around the school at a depth where the wind induced underwater toroidal motion in the vertical plane becomes negligible such that the flying fish is unable to sense the tailwind direction above, limiting the ability to swim up and emerge in the right direction to glide. Experiments confirm that the flying fish tail rigidity is too low for a quick ballistic exit, which is not called for either.

## Introduction

Due to photosynthesis, the top layers of the tropical ocean abound in life forms and predator-prey interactions (defined in "Methods" section). In the predator-prey interaction of sailfish-flying fish captured in the vivid videographies of Attenborough^1^ (and https://drive.google.com/file/d/1gn-uobapyDTq7DYlEkmlRuEBC7ExYxA2/view?usp=drive_link), at the time stamp m:s during 0:45−0:51 ("Methods" section), an easy to miss highly organized small surface wave packet appears on the free surface, dispersing radially while maintaining the coherence. Where does the wave packet come from and why is it formed? Furthermore, near the free surface, the ocean is semi infinite. Hence, how does a sailfish corral about a hundred flying fish single handedly thwarting their escape up for gliding, or down in the deeper ocean? (A second sailfish sometimes joins-in, but later). While the sailfish is remarkably successful in corralling, why does it equally remarkably fail to capture hardly any flying fish despite active pursuit? The latter is more surprising because a sailfish is a tertiary consumer–an apex predator, while the flying fish is a secondary consumer. A theoretical interaction model is given that explains how the wave patch is formed and why the corralling is first so successful, but later an uncommon topological bifurcation instability allows the prey fish to escape.

A critical context of the interaction is that the sailfish initially establishes 1 m as the length scale while the flying fish length scale is 0.1 m, their body lengths to which the cruising velocities are related. The most remarkable aspect of the interaction lies in the school topology and an instant of time suddenly appears when the school compacts and collapses asymptotically to a ‘point’ whereby the flying fish school, instead of swimming parallel to each other, swim collectively to a virtual origin as in a sink flow, with gaping mouths in seeming panic. Physically, the interaction scale diminishes from 1 m $\rightarrow$ 0.1 m. Because schooling, which is related to fear, is as deep as evolution, what could have overridden such a basic instinct? The topological instability and the trigger of the attendant fear are modeled as if sourced by an acoustic impulse which ultimately acts on the flying fish brain causing unbearable pain. The kinetic energy of the vortex rotation is abruptly halted by the sailfish to create a pressure impulse which reverberates between the oppositely placed sailfish and the flying fish school which act as concave acoustic mirrors. Euler wave and Lighthill noise equations are used to compare the theory with the free surface wave footprint of the acoustic event. A vortex breaking model is given to estimate the pressure and time scales of the impulse.

**Figure 1:**
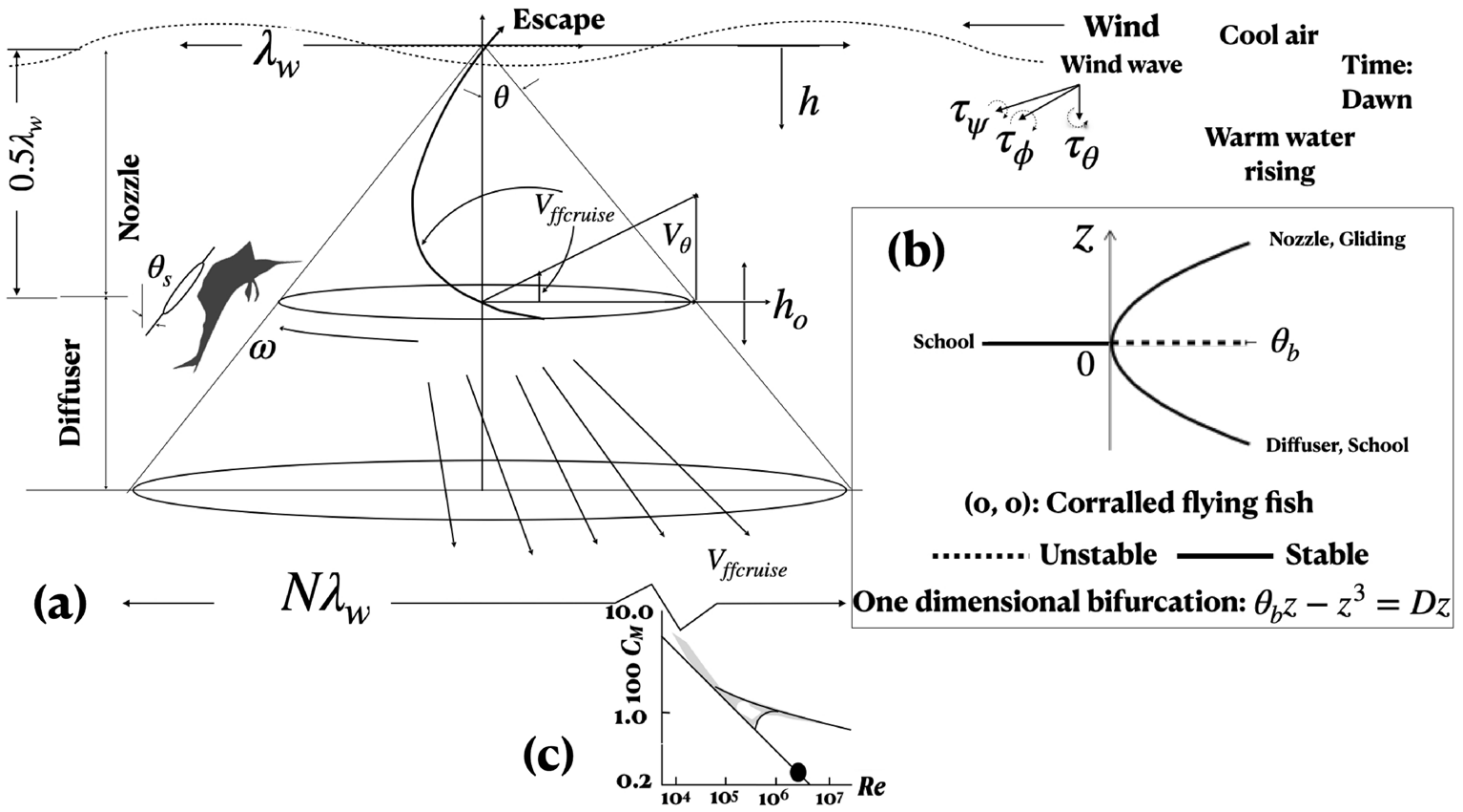
The sailfish vortex (**a**), the interaction model (**b**) and the sailfish coefficient of moment in the swim plane (symbol, **c**). In (**a**), the flying fish school (not shown) is situated opposite to the sailfish. The oppositely placed concave and vertically tilted sailfish and the flying fish school in the swim plane constitute the cloak. (**a**) Boundary-layer classification of the vortex into nozzle and diffuser flows. The sailfish is vertically tilted at angle $\theta _s$. (**b**) Topological instability of the flying fish school; first a singularity is formed when the flying fish motion comes to a halt at $\theta _b = 0$, $z = 0$, then two branches are formed; in the upper branch, individual flying fish escape up along spiraling streamlines in the nozzle; in the lower branch, the school reforms, swimming below, in parallel, in the diffuser. In the swimplane ($z=0$, $h=h_0$ for both predator and prey), the rapidly declining velocity *U* and separation $\theta _b$ of sailfish dominate the interaction pre-bifurcation from $-\theta _b$ to 0. The close-range zig-zag motion of interaction is intense in the immediate post-bifurcation near $\theta _b = 0$. The sailfish does not interact later post-bifurcation where $\theta _b > 0$ and for flying fish $z > 0$ or $z < 0$; the sailfish remains in the swimplane thereby increasing the separation. The flying fish cruising returns where $z > 0$ or $z < 0$. The interaction then is about reduction of swim velocity and separation−a frictional process. The concave sail fish and flying fish bodies cloak (wrap around) the space of vorticity and acoustics. (**c**): shaded area is laboratory disk measurements, left line is laminar, right line is turbulent and the curved line is transitional.

The interaction takes place at dawn, when the sun is rising in the horizon and has not started heating the surface. There is a breeze but it has not picked up stirring waves which interfere with flying fish taxiing. The predatory frigate birds have not yet appeared. In this brief time window of the day, the conditions are right for the flying fish to emerge, taxi on the surface and glide, which they do in the same direction hinting at cost minimization. But, in an interaction, why doesn’t the entire school glide away when frigate birds are yet to appear? An awareness of the tailwind direction while still underwater can be expected, but only up to a depth. The sailfish undertakes two kinds of motions: an initial steady circling of the school and then a one-to-one close and active zig-zag pursuit. A mere half a turn of steady circling appears to trigger the spontaneous rotation of a vertical vortex (Fig. 1). Because the sailfish span < length, the induced drag is high and yet, the speed does not drop during the zig-zag motion. Hence, how is the sail aspect ratio actively managed to keep the drag low? The vortex defines the control volume and entraps the school. The spontaneous start of the vortex suggests the presence of a critical Reynolds number and the sailfish circling provides the critical rotational velocity. The vortex energy is primarily supplied by the thermal stratification, to be explained below. The diverse mechanisms have been synthesized.

Applying Newton’s law of friction, the shear stress $\tau = \mu \partial V_\theta / \partial z$, where $\mu$ is the absolute viscosity of water, $V_\theta$ is the circumferential velocity and *z* is the vertical coordinate. Imparted by the sailfish, $V_\theta$ is a maximum in the swimplane and drops to zero at the free surface, the friction making the vortex conical (Fig. 1a). Minimizing body friction, the sailfish has a tilt which helps it to keep an eye on the school and the constriction of the cone limits the flying fish escape. The tilt angle $\theta _s$ is a measure of Newton’s shear friction. Notice how the Newton’s friction-law, which is stabilizing, is bounding the interaction to lead to a topological collapse, an acoustic focus and the formation of a monopole. Friction governs the entire sailfish-flying fish interaction, but destabilizing inertia force does not. Indeed, an instant of time exists when the fish motions come to a halt and a singularity is formed (Fig. 1b)^1^. The forked nonlinear bifurcation of the topological instability leaves a rare room for the not-too-small fish to escape the large, in droves, in spite of being corralled. Question remains, what is the depth that the sailfish selects for the planar interaction? The depth defines the vortex angle and the work done, which must be a minimum. It is known that a horizontal wind over a free surface creates a toroidal rotation in the vertical plane below^2^. If the wind-induced wavelength of the surface wave is $\lambda _w$, then the toroidal motion drops to zero at $0.5 \lambda _w$, as shown in Fig. 1. The flying fish would be unable to sense the wind direction if corralled at a minimum depth of $0.5 \lambda _w$. Corralling at a smaller depth would increase the number of flying fish gliding away, and a higher depth would increase the vortex mass and work done. During the daytime, solar radiation heats the ocean surface which gradually heats the water below. At night, the warm water below rises and the sea breeze cools the surface. Vertical thermal stratification exists at sailfish length scale which inverts in 24 h. An analogy may be drawn with hurricanes which spontaneously start when wind speed reaches 33 ms$^{-1}$ and there are thunderstorms present in the neighborhood due to heating of the earth’s surface and the presence of moisture. In the sailfish vortex, the critical velocity is probably about 1–10 ms$^{-1}$, the cruise velocity range.

Through molecular transport, non-uniformities in the intensities of heat and momentum are eliminated by their kinematic diffusivities $k_H$ and $\nu$ (m$^{2}$s$^{-1}$) which are analogous^3^. As a result, the diffusion equation for a quantity $\Phi$, such as momentum, heat and concentration, is the same, namely, $\partial \Phi / \partial t = k \nabla ^2 \Phi$, where *t* is the time and *k* is the diffusivity. The flux $F= - k \nabla \Phi = - k \nabla T$, where *T* is temperature. This molecular mechanism converts thermal stratification into a vortex when critical conditions are reached. The transport of heat and vorticity for Prandtl number of one is analogous. This principle can be applied to the energy equations in axisymmetric boundary layers, such as a vortex^4^.

In the vast area of research of predator-prey interactions^5^, where the interest is frequently in the context of disease vectors^6^, field evaluations of oceanic interactions are almost always rare. The interaction in the evolutionary time scale is generally in agreement with equilibrium theory although the stability is difficult to evaluate^7^. In terms of marine ecosystems, the present work is concerned with the biology-inspired hydrodynamic engineering aspects of the direct interaction of limited time scale (s) between a tertiary consumer, a sailfish-an apex predator, and a secondary consumer, a flying fish^8^. Sailfish feed on shrimps, squids, sardines and mackerel, and normally not on flying fish. Flying fish have an upwardly cambered profile, can penetrate the free surface, taxi on the ocean surface and then glide in air. Comparatively, sardines are smaller (0.125 kg vs. 0.91 kg) and mackerels lack wings and are not cambered. Bottom-dwelling flying gurnards can measure up to 0.50 m long and can glide above water or walk on the seafloor using their fins^9^ . Nearly matched in circulation, in contrast to whales and krill, the sailfish’s ability to corral an entire flying fish school is remarkable, and despite the school’s success in thwarting capture, the manner in which a few flying fish break from the group to swim upwards to breach and glide is explained as a topological instability. Anthropogenic noise disrupts antipredator behavior^10–14^. Fish predation can increase by anthropogenic noise such as continuous motorboat roar^14,15^. But the triggering of a natural, transient, acoustic phenomenon in the ocean and then the use of the reflective body curvature to focus and amplify the noise on the target prey to disrupt their motion does not seem to have been reported. For a mass ratio of 0.01, the initial interaction is between a school of the secondary consumer and an individual tertiary consumer. For mass equivalence, the school size is 90. While the sailfish has a remarkable energy saving strategy of corralling, the strike rate is a low 2% and the capture rate is a mere 1% in an interaction duration of 18 s^1^.

This paper explains how, in the context when the flying fish is not a common diet of sailfish and their circulations $\Gamma$ are not widely differentiated ($mg = L = T = D = \rho U \Gamma$), where *m* is mass, *g* is acceleration due to gravity, *L*, *T*, *D* are lift, thrust and drag forces, $\rho$ is density of water and *U* is fish velocity, the sailfish−although an apex predator, uses the energy-saving contact-less mechanism of acoustic reverberation to disorient the flying fish brain, albeit temporarily, to collapse and corral the school. That a school can be collapsed is surprising. The mid-ocean hydrodynamics and hydroacoustics are interesting components of presently unavailable large predator-prey interaction modeling. A theoretical model is given synthesizing the diverse mechanisms. The observed surface wave patch is also compared with the Lighthill monopole noise simulation^16^. Experience shows that in autonomous (self-driven) multi-scale flows, when one mechanism is unstable (a bifurcation is present)^17^, or rather multistable^18^, then the other mechanisms are so as well. Hence, experiments on air-sea interface instability and flying fish tail hardness are carried out to determine drag (= thrust), measured indirectly by interface Weber number. Crowns and spikes, characteristic of transitional Weber number *We* are present when the flying fish emerges from underwater ("Methods" section)^1,19^. The fish tail hardness experiment also has to have similar Weber numbers and it indeed does.

## Results

### Effect of bending moment on fish mobility

The ratio of the half major to minor axes of the elliptic cross sections are $a/b$ = 1.71 and 2.90 in the flying fish and sailfish, where $a$ is along the vertical axis $x$, and $b$ is along the horizontal axis $y$. The moment of inertia are $I_x = (1/4) \pi ab^3$ and $I_y = (1/4)\pi a^3 b$. Thus, $I_y>> I_x$ in the sailfish, but $I_x \approx I_y$ in the flying fish allowing the former to camber easily in the horizontal plane while the latter can apply torsion. One-to-one pursuit shows torsional escape by a corralled flying fish below the swim-plane^1^. The sailfish then is a planar swimmer while the flying fish is a three-dimensional swimmer. Because the smaller flying fish swim in schools, it is easier to corral them in the horizontal plane. Assume $\pi d = 2L$, where *d* is the minimum packing diameter of the school and *L* is the length of the sailfish. For *L* = 1 m, $d =$ 0.64 m. If $d=20 b$, $b =$ 3 cm, which is reasonable, that is 10 flying fish are stacked side by side. We get $10^2$ fish in the school which is approximately as observed^1^. Alternatively, for a 50 kg sailfish, the equivalent flying fish mass is 0.50 kg which is reasonable. Approximately, the packed flying fish school equates to a sailfish.

For a sailfish length of 3 m, mass of 100–200 kg, and a flying fish length of 0.2–0.45 m, mass of less than 1 kg, the flying fish to sailfish mass ratio is approximately 0.01, and the length ratio is 0.1. Sailfish are a sink of fuel (J, a tertiary consumer) that renews mass in a certain time period. The metadata for cruising, over eight logarithmic decades, from large engineering swimmers to animal swimmers from sharks down to bonito, follow the same trend of propulsive power *P* (kW) versus displacement volume (m$^3$), namely the output power $P=TU = CL^3$, where *U* is cruise velocity-a constant for a particular swimmer, *L* is a length scale and *C* is a constant^20^. Writing $T=\rho U \Gamma$, $\Gamma =L^3/U^2$. The ratio of the cruise circulation of the sailfish to that of the flying fish $\Gamma _s/\Gamma _{ff} \propto (L_s/L_{ff})^3 (U_{ff}/U_s)^2$. For $U_s/U_{ff}$ = 10, and $L_s/L_{ff}$ = 10, $\Gamma _s/\Gamma _{ff}$ = 10-not all that higher. How big is the area of influence of the sailfish? From Biot-Savart law for a two dimensional vortex filament, the circumferential velocity $V_\theta = \Gamma _s/(2 \pi r)$, where *r* is the distance normal to the vortex axis. Therefore, $V_{\theta s} = V_{\theta ff}$ at a distance from a sailfish of $r_s$ = 10 m near, say, 1 m from a flying fish. At dawn, when the mid-ocean background SPL (sound pressure level) is low (= 70 dB), the sailfish creates oscillations in an area of 100 m$^2$ just beneath the free surface. Corralling starts when the distance between the sailfish and the flying fish school is induced to drop below 10 m.

### Spontaneous start, instability and bursting of a sailfish vortex and the creation of a monopole

**Figure 2:**
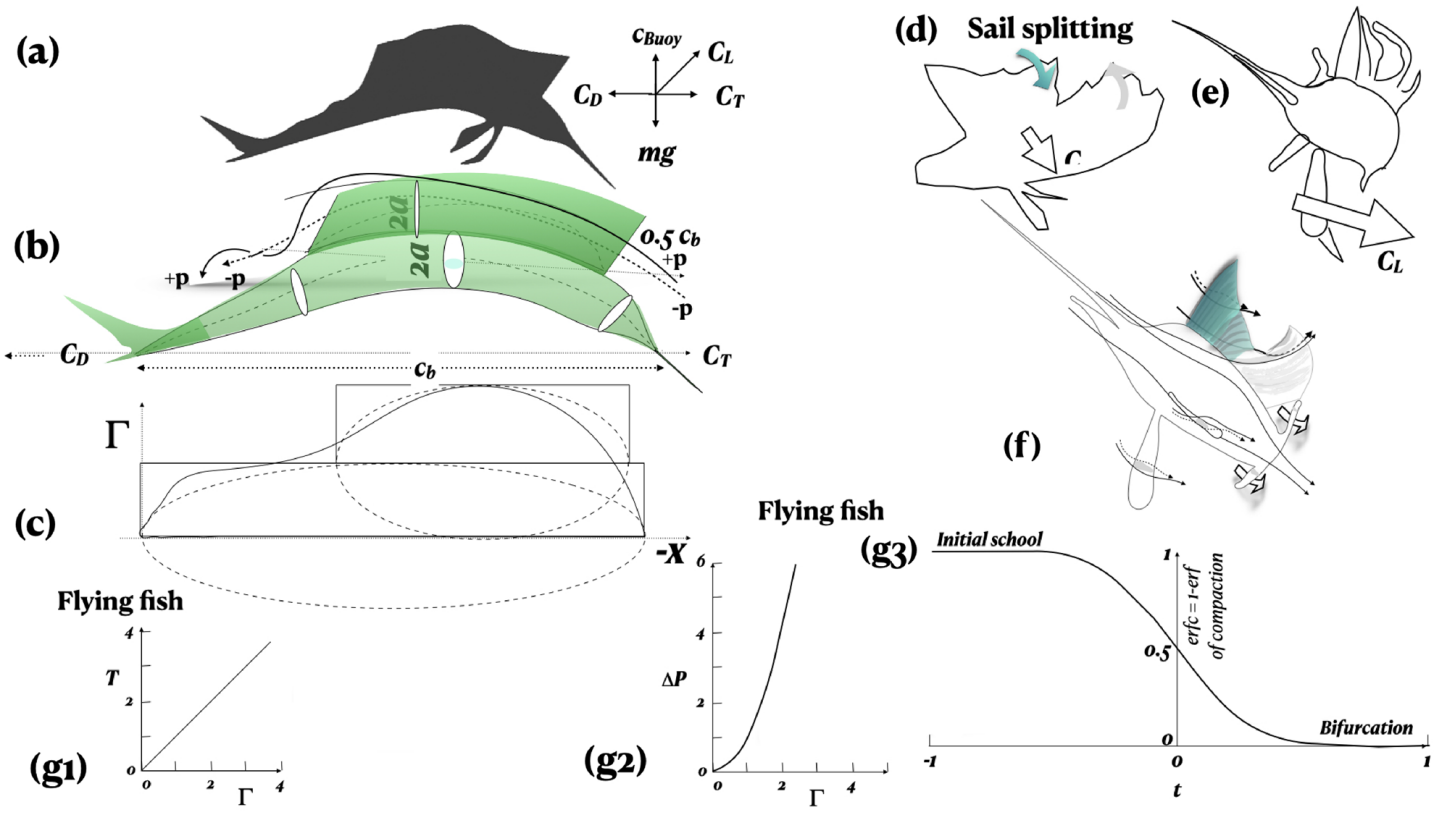
Circulation $\Gamma$ model of thrust (**a**–**c**) and winglet model of control of induced drag (**d**–**f**) of sailfish^1^. (**a**) Force diagram: coefficients of lift $C_L$, thrust $C_T$, drag $C_D$ and buoyancy $C_{Buoy}$ and *mg* mass force. (**b**) Chord $C_b$ and span $0.5C_b$; elliptic sections shown; pressure ($+p$) and suction ($-p$) side streamlines shown. (**c**) Circulation $\Gamma$ distributions, curved solid line; broken lines: elliptic distributions of $\Gamma$ due to main body and sail; solid boxed lines: two-dimensional idealizations. The sailfish is front loaded as in small two-dimensional wing training aircraft. Continuous lateral sail oscillations during close pursuit occur at the center of pressure. (**d**, **e**) Dorsal sail splitting and winglet formation: long span and smaller chord. (**d**, **f**) Two winglets. (**e**) Multiple winglets. (**f**) Notional chord-wise streamlines; winglets reduce spanwise flow and lower induced drag. (**d**) Splitting; (**f**) merging back. Time increases (**d**) to (**f**) during slow down. Sailfish streamlines are always axial−during cruising as well as maneuvering. (g1–g3) Modeling of flying fish monopole formation; g1: time scale *T* of vortex breaking versus circulation $\Gamma$; g2: pressure $\Delta P$ released by vortex breaking versus circulation $\Gamma$; g3: compaction of flying fish vortex school due to diffusion versus time *t*; dimensional estimates in text; school compaction makes the shed propulsion vortices touch, break and radiate the pressure of the centrifugal rotation creating a monopole.

**Figure 3:**
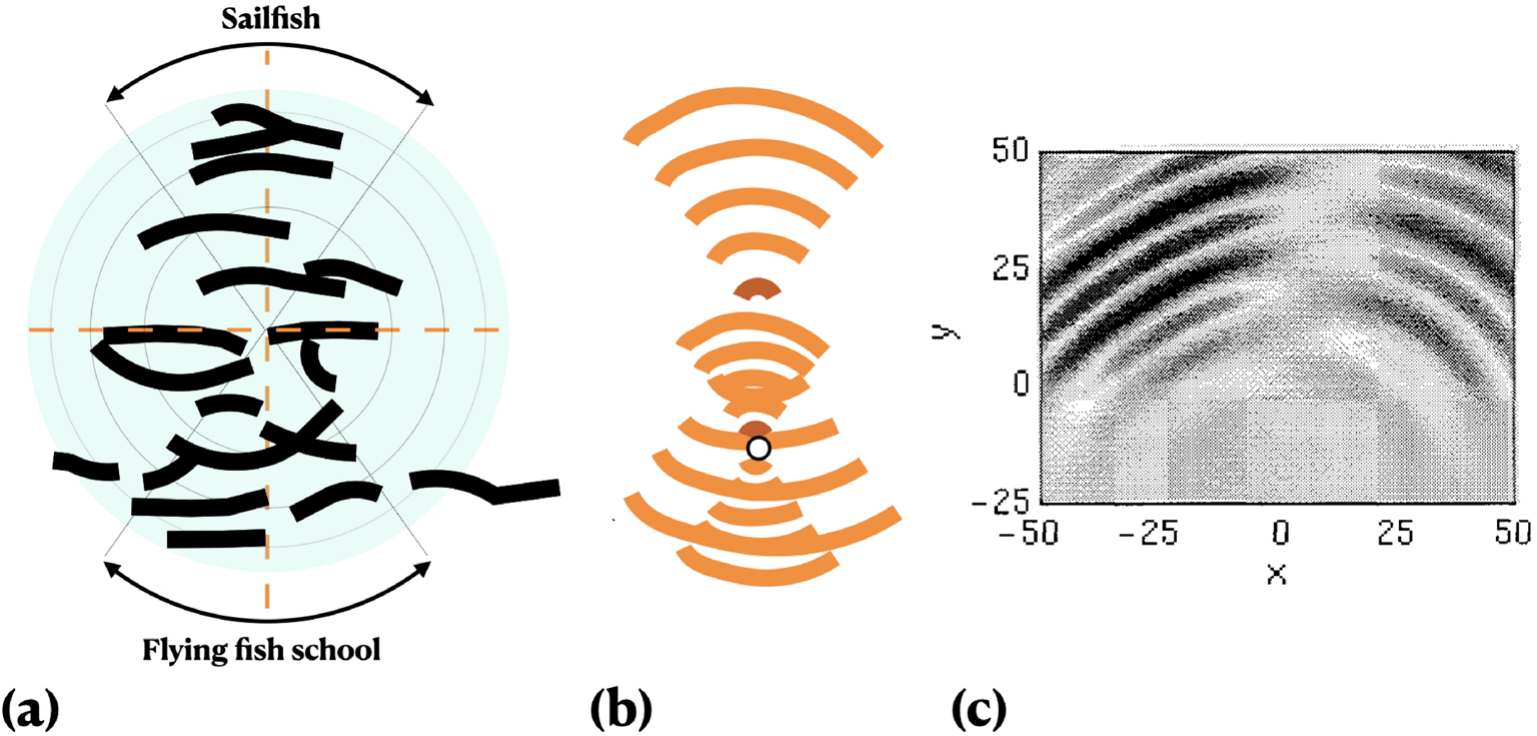
Acoustic predation map. (**a**) Free surface wave cluster (observation). (**b**) (0.5$\lambda$ + $\lambda$) wavelength superposed qualitative model (present work). O: flying fish singularity. (**c**) Results^16^ of monopolar source pressure field computation of Euler–Lighthill equation; the ‘quietened’ horizontal radial alleyway in (**b**) is present here; figure reproduced with permission of authors^16^.

The solar and wind effects on the top ocean layer mentioned before, create a vertical thermal gradient and an axial flow of velocity $V_a$. Bounded by the free surface and the swim plane below, the circling of the sailfish at the critical $V_\theta$ about the oppositely placed flying fish school creates a conical vortex similar to hurricanes and rotating disk boundary layers (Fig. 1, "Methods" section). The scales of the rotating flow are *L*/*D*, the ratio of the axial length to the swim-plane diameter and $V_\theta /V_a$-a proxy for Taylor number (Fig. 2 in^21^). Visualization in centrifugal gas-liquid separators^21,22^ shows that the vortex core oscillates between straight and spiral in certain combinations of *L*/*D* versus $V_\theta /V_a$, as documented in the stability $\pi$-lines in Fig. 3 in^21^. The NOAA videos of Atlantic hurricanes also clearly show an interaction of the horizontal outer spiralling bands and the vertical core. As the sailfish spirals inwards, the core rotates faster and tightens. When $V_\theta$ drops suddenly, say by the sailfish stopping, the vortex would burst due to a sudden change in cross sectional area^3,21,23^. The oscillations may induce the bifurcation in Fig. 1b.

### Dividing the sailfish vortex into nozzle and diffuser

The sailfish vortex in Fig. 1a is modeled as a boundary layer on a rotating disc submerged at ($h_o$) demarcating a spiralling nozzle flow upward and a radial source flow downward. The vortex scales to the wavelength $\lambda _w$ of the wind waves setting up an orbital (rolling $\phi$) motion underneath the free surface by applying a rolling torque $\tau _\phi$: the wave base depth is $0.5 \lambda _w = h_o$ where the swim−plane is located and for integer *N*, the diameter is $N \lambda _w$.

The sailfish pitch $\theta$ torque is $\tau _\theta$. The coupling of the orthogonal torques $\tau _\psi =$ $\tau _\theta \times \tau _\phi$ yaws $\psi$ the vortex axis slightly because $\tau _\theta<< \tau _\phi$ (Fig. 1 inset near the free surface)^23^. In the vertical shear $\partial U/ \partial y$, the tilt increases due to induced velocity, axially stretching the vortex while reducing its diameter and compacting the school. The vorticity is enhanced. The positive feedback loop continues until the tilt angle reaches 45$^{\circ }$, the direction of principal strain, when the vorticity reaches the maximum value. Further stretching bursts the vortex releasing its energy^3,23^. Replace the circling sailfish by a horizontal disk, of zero thickness, of swim-plane diameter at $h_o$, rotating at the same angular velocity $\omega$. Apply boundary layer approximations to the Navier-Stokes equations in cylindrical coordinates $r = R, \theta , z$ and velocities $u_R, u_\theta , u_z$^2,24^. Two domains are produced (Fig. 1): (1) where $0< h < h_o$, the three forces are assumed to be roughly of same order, as commonly is the case in oceanic systems, albeit which tend to be large $F_{Eul} \approx F_{Cen} \approx F_{Cor}$; and (2) where $h_o< h < h_\infty$, $F_{Eul} \approx F_{Cen}, F_{Cor} \approx 0$. Here, $F_{Eul}$ is the Eulerian force, $F_{Cen}$ is the centrifugal force and $F_{Cor}$ is the Coriolis force. For $0< h < h_o$, $u_R= u_\theta = u_z = u$, and for $h_o< h < h_\infty$, $u_z = u_R = u$ and $u_\theta = 0$. For $\partial / \partial t = 0$, where *t* is time and for inviscid flow, the boundary layer equations reduce to simplified forms. The rotating disk splits both the vortex flow and the flying fish school in the swim plane.

For $0< h < h_o$, $\partial p/\partial R = \partial p/\partial \theta = \partial p /\partial z$. Hydrostatic pressure $p \propto h$. The boundary-layer solution of the streamlines is a spiraling flow^24^. The flow directions are reversed whereby it also matches hurricanes. This outcome satisfies the elastic property of a vortex, namely that in a constriction, a spiral will result^3,23^.

For $h_o< h < h_\infty$, $\partial p/\partial \theta = 0$, forces $f_R = f_\theta = 0$, $\partial /\partial \theta = 0$, $\partial u_z /\partial z = 0$. There is no flow downward and the schooling resumes. The simplified equations are: $\rho u_\theta ^2 / R = \partial p / \partial R$, $u_R ( \partial u_\theta /\partial R)-(u_\theta u_R)/R=0$, and $\partial p/\partial z = 0$ (this *p* gives vortex turning effect excluding static head). There is no vortex cross-sectional area change and spiraling. The simulations agree with the observations of flying fish trajectories.

The human swimming rate of energy expenditure is 0.83 kJ/s. For a flying fish capture rate of 1%^1^, a 100 g flying fish offers 1700 kJ, the sailfish needs to spend only 10.2 kJ in 12 s, the duration of pursuit. Unskinned-dried tadpole fish meal offers 1639.63 kJ/100 g, that is, 2/3rd of terminte meal, the highest value^25^. This is 0.85 kJ/s, which is close to the human swim energetics rate. The low capture rate therefore implies a low work done rate–another evidence that the interaction is friction, not inertia driven.

### Sailfish coefficient of moment

For a rotating disc of radius *R* and diameter *D*, wetted on both sides, the moment is $2M = 0.616 \pi \rho R^4 \sqrt{\nu \omega ^3}$ (Fig. 1c). Take kinematic viscosity $\nu$ = 10$^{-6}$ m$^2$s$^{-1}$, $\pi D$ = 15 m, disc *R* = 2.4 m^24^. Sailfish *V* = 1 (ms$^{-1}$)^26^, one rotation of the sailfish takes 15 s, $\omega$ = 0.42 rads$^{-1}$. Then, 2*M* = 17.48 Nm. The free surface wave footprint of the vortex below dissipates in $\ge$ 5 s, which is the sailfish rotation rate. The coefficient of moment $C_M$ = $2M/(0.5(\rho \omega ^2 R^5))$ = 0.0025. Reynolds number $Re = R^2 \omega / \nu$ = 2.42x10$^{6}$. For laminar flow theory, $C_M$ = 3.87/$\sqrt{Re}$ = 0.0025 . Hence, the sailfish moment is below all laminar and transitional engineering rotational solid disc flow data (Fig. 1c)^24^.

When the hydrodynamic force exceeds the bending force, the sail ray oscillates. Estimate the frequency $\omega = 2\pi f$ of spanwise oscillation by equating the two forces: $\omega = \sqrt{(\rho _{w1}U^2 /(\rho _{w2} h L )}$^27^. Assume all densities to be the same as of water. Say the average thickness of the sail *h* = 0.01 m, wing span = *L* = 0.1 m, and velocity *U* = 1.0 ms$^{-1}$. These values give $\omega$ = 31 rads$^{-1}$, giving *f* = 5 Hz for spanwise fluttering. The webbing would lower *f*. For a dorsal fluttering, *f* = 2.2 Hz is present. This frequency is in the range of dolphin fluke oscillation measurements^28^. The tail beat frequency of the flying fish is 0.5—1.0 Hz. For thrust $T \propto f^2$, the flying fish *T* = 0.25–0.5 of the sailfish *T*^29,30^. For a faster chase if *U* doubles, *T* would have to rise by x16-a prohibitive rise, limiting the flying fish planar mobility. Hence, when the need for higher escape speed comes, the flying fish would twist and swim off-plane instead, as indeed the video shows^1^.

### Sailfish winglets: spanwise flow control

The cambered sailfish acts as a two-dimensional wing, having elliptic circulation $\Gamma = \Gamma _o \sqrt{1-(x/(0.5b))^2}$, where the span is vertically oriented^31^. The finiteness of the span requires spanwise flow control to lower induced drag $C_{di}$. The sails add about half of the local $\Gamma$ in the front half, and as a result, are used to stabilize the main body (Fig. 2).

Counterintuitive to low speed engineering (Fig. 2), the sailfish chord $\times$3 of the span increasing $C_{di}$. The sail doubles the local circulation $\Gamma$ and has negligible thickness *b*. The sail can split in up to four winglets to prevent the streamlines from moving toward the sail tip lowering $C_{di}$.

During frequent turning, the sail splits into two sails creating two leading edges (Fig. 2d, "Methods" section). Azimuthally, the front sail tilts inward to the pressure side of the cambering and the rear sail tilts outward to the suction side of cambering producing two contra-rotating wing-tip vortices. Due to cooperative instability, the two oppositely rotating wing-tip vortices braid downstream lying in the horizontal sailplane. This braided vortex pair ensures the vertical division of the corralling vortex into the nozzle and the diffuser segments. The angle of attack is high and momentum dissipation neglected due to short trailing distances and times. The wing-tip vortex core reduces the jet-like induced drag^31^. The rear sail is tilted. The chord is reduced compared to the straight sail, the span remaining unchanged.

The drag coefficient of the sailfish is $C_d = C_{do} + C_{di}$ , where $C_{do}$ is the base drag coefficient of the uncambered sailfish at zero lift. Neglect viscous drag. Here, $C_{di} = C_L^2 / (\pi A_R e)$, where $C_L$ is the coefficient of lift, $A_R$ is the aspect ratio of the winglet and *e* is the planform efficiency. Further, $A_R = s^2/A$, where *s* is span and *A* is the winglet area. The sail and the winglets are rectangular, making $A_R = s/c$, where *c* is the chord. In the sailfish, $s =$ constant and *c* is reduced to change $A_R$. As per lifting line theory, $C_{di}$ is the lowest if $\Gamma$ distribution of *L* is elliptic whereby $e = 1$^31^. Animals are optimized (Fig. 2c). For $C_L =$ constant, $C_{di} \propto 1/A_R$.

Say the sail splits into two equal area winglets of same span *s* and of chord $c_1 = c/2$. Say, winglet lifts are equal $C_L = 2C_{L1}$. Then, $C_{di1} = C_{L1}^2 / (2 \pi s/c)$, $C_{di1} = (C_L ^2 /4)/(2\pi s/c)$ = 1/8$C_L^2/(\pi s/c)$. Two winglets have a total induced drag of $C_{ditot} = (1/4) C_L^2/(\pi s/c)$. Hence, the induced drag is reduced by a x4. The $s/c = 1/3$ in the undivided sail, but $>>1$ in the winglets. The wide winglet portfolio means that the sailfish reduces $C_{di}$ at all speeds. Methods gives the properties of the axial locations of the two primary winglets $W_1$ and $W_2$, where the streamlines and circulation gradients change sign in order to improve stability. In Fig. 2d–f, the winglets are deployed then merged back as the camber $\rightarrow$ 0, and $U \rightarrow 0$. The sequence is similar to bald eagle landing.

The near-stall sail lift $C_{Ls}$ is enhanced for the following reasons. (1) The proximity of $\Gamma _1$, $\Gamma _2$ increases the angle of attack increasing $C_{Lmax}$. (2) A new thinner boundary-layer develops in $W_2$ allowing it to delay separation. (3) The incoming flow to $W_2$ is from the suction side of $W_1$ which lowers pressure spikes ($C_p$) at the leading edge. The $C_p$ peak is also reduced because $\Gamma _1$ lowers $\Gamma _2$. The leading edge is thinner in $W_2$ than in $W_1$. (4) However, $\Gamma _2$ increases $\Gamma _1$ improving the performance of $W_1$. (5) A large width of the intervening alleyway allows the wake of $W_1$ to expand away from the boundary-layer effects of the pressure side of $W_2$. (6) The opening and closing, large span alleyway keeps the streamlines parallel to the swimplane, away from spilling over across the tip. A two-dimensional flow is produced and $C_{di}$ is reduced (negligible downwash).

### Vortex footprint on the free surface

A cluster of waves on the free surface appears when the individual flying fish begin to glide in air, all in the same direction. The cluster is composed of a group of waves whose radial spacing is an order of magnitude smaller than the spacing of the surrounding wind-induced waves. In  5 s, the cluster expands radially, maintaining their phase while merging with the background. The wave cluster is modeled as the free surface cut of a transient acoustic event located beneath the sailfish swimplane, demonstrating spherically radiating energy. The apex of the vortex, a stagnation point, is quiet because inertia force $\xrightarrow {}$ 0 and stabilizing pressure and viscous forces balance: $pL^2/\rho \nu ^2 = 1$, where *L* is a capillary wavelength ($\le$ 0.007 m), *p* is the pressure, $\rho$ is the density of water and $\nu$ is the kinematic viscosity. When *L* $\le$ 0.007 m, surface tension restores flatness^32^. The larger wavelength constant phase rays in Fig. 3(a,b) are therefore gravity waves where inertia force is the destabilizing force, but both surface tension and gravity waves are stabilizing forces. The frequency of the wave is $f^2 = k(g + Tk^2/\rho )$ , where *g* is the acceleration due to gravity, *k* is the wave number and $\rho$ is the density of water. If the first term on the right dominates, the effect of gravity is stronger. If the second term dominates, the effect of surface tension is stronger (Fig. 1d in^23^).

### Corralling of flying fish

#### Flying fish topological instability and singularity formation

Unexpectedly, the sailfish−a fast swimmer, uses the stabilizing mechanism of friction to halt all motion as the means of predation and not the destabilizing inertia force.

In a school, say the individual fish $+\Gamma _f$ or source strength form an ‘elastic’ network of nodes of position vector spacing $\Delta r_f (t)$ of equal magnitude. The equilibrium spacing is disturbed impulsively via Biot-Savart induction when a sink or $-\Gamma _s$ is first seeded where $| \Gamma _s |>> | \Gamma _f |$ resulting in $\Delta r_f (t) \rightarrow 0$ -an irreversible, topological and unstable singularity forms whence at least five fish turn simultaneously inward toward a point ("Methods" section)^1^. To disturb the equilibrium to induce a topological instability, the sailfish suddenly starts swimming in the counter direction nullifying the induced oscillations in order to still the water. There is evidence that the sailfish motion then is opposite to the school^1^. The instability is modeled as a one dimensional pitchfork instability given by $Dz = \theta _b z - z^3$^33^. The steady state solutions for $\theta _b < 0$ and $\theta _b > 0$ are shown in Fig. 1b where the corralling singularity is located at $\theta _b, z = 0$. Post-bifurcation, two stable branches are possible. In the lower branch, most of the fish restore the school to swim below the swimplane in the diffuser (Fig. 1). In the upper branch, a few individual fish swim up to the nozzle, breaching the interface in order to glide (Fig. 1).

#### Modeling of the monopole formation mechanism

To create the monopole, seed the swim plane with propulsion vortex pairs. Say the sailfish flaps the tail just enough number of times to encircle the school and vanish. Each flying fish flaps only once and vanish. Hypothesize the approach to the school bifurcation (0, 0 in Fig. 1b) proposing that each flying fish and the encircling sailfish tail oscillation produces a pair of vortex circulations $\pm \Gamma _f$ and $\pm \Gamma _s$, where $\Gamma _f<< \Gamma _s$. The vorticities $\omega$ of the vortices contain centrifugal force which is released as pressure $\Delta P$ when the vortices touch and break^34^. The like signed circulations pair^35^. The distribution in the school of flying fish vortices is compacting and tangling (touching initial condition). The strain makes the vortex cross section elliptical, tending to flat. There will be stretching of the curved length yielding more locations of principal strain than when straight. The time scale of breaking is $T = C/\Gamma _f$, where the constant *C* is a function of the radius of curvature of the tangle and initial vortex diameter when unstrained (Fig. 2g1). The pressure release of breaking over depth pressure is $\Delta P = \rho D \Gamma _f^2$, where *D* is a function of the vortex elliptic cross section (strain makes the cross section elliptic from a circular core initial condition) and strain, and may be assumed to be a constant (Fig. 2g2). Until the bifurcation, the negative feedback compaction is a diffusion process, given by $\partial \Phi / \partial t = k\nabla ^2 \Phi$, where *t* is the time and *k* is the diffusivity, which can be described by an erfc (Fig. 2g3). More the school and the vortices compact, slower the diffusion becomes, until unstable. Say, the normalized initial school area *A* is 1.0 and the compacted area $(1-A) \rightarrow 0$ when bifurcated. The compaction process is $\dot{\delta }= \beta (1/\delta )$, where $\beta$ is a constant.

Vortices touch when the school is compacted. Model the pressure of the monopole from the breaking of touching vortices $\Delta P$ (N/m$^2$) = $\rho D \Gamma _f^2$, where $\rho$ is density, the geometric parameter *D* (m$^{-2}$) = $f(\theta )/(2 \pi ^2 bh)$, the axis ratio $\theta = h/b$ and $f = \theta /(1+\theta ^2)$. Say, the highly stretched and paired flying fish tail shed vortices, when compacted (Fig. 2g3), are touching and breaking, and are similar in geometry to the colliding ring vortices in the laboratory ($\Gamma$ = 16 cm$^2$/s)^34^, but has $\times$400 higher circulation . Here, *h* and *b* are the semi elliptic axes parallel and normal to the line joining the vortex centers. The pressure $\Delta P$ is 1.66 kPa, comparable to the earlier 1.4 kPa estimate from the surface waves. The vortex breaking time is circulation-ratioed to be 0.0018 s from touching. This time equals the post-monopole first reverberation time estimated below from Fig. 3a.

#### Cost of the underwater acoustic event

The conical vortex (Fig. 1a) forms spontaneously when the sailfish turning velocity $V_\theta$ reaches the critical Taylor number $Ta_c = V_\theta / V_a$, where $V_a$ is the axial velocity. The $V_\theta$ creates radial and vertical pressure gradients leading to a large-scale circulation. A positive feedback loop is setup: as $V_\theta$ increases in the swim plane, the vertical upward spiralling flow in Fig. 1 increases drawing more of the vertically stratified thermal energy, increasing $V_\theta$ further. Receiving thermal input, the inertia force of the circulation is large compared to the sailfish input. The free surface acts as a vertically compliant solid wall. The sailfish makes half a full turn to trigger the vortex formation^1^.

The sudden stopping of the sailfish is analogous to a glass of water firmly struck on a table when radial surface wave modes radiate from the center; Methods^2^. While the potential energy ($\rho g h$) is not changing because $h = h_o$ is a constant, the kinetic energy of the fluid of mass *m*, $0.5 m v^2$ and velocity *v*, changes to pressure energy when the vortex churning is stopped ($v \rightarrow$ 0). The pressure impulse energy radiates spherically. Some energy is reflected by the concave sailfish and flying fish bodies, going back and forth creating standing waves (Fig. 3). The *p*-impulse reverberates in the flying fish bladders causing putative pain, disorienting the flying fish sense of direction^36–38^.

The Taylor exponential time history of *p*, the peak pressure pulse ( 0.2 psi at $h=0$, estimated from wave height, Methods^1^, Fig. 3) of the energy release from the vortex and the distance of the fish from the monopolar source ($\le 2.5$ m) gives the sound pressure level (SPL dBm re. 1 mW). Heat dissipation in salt crystals, and losses where there is no reflector, can be neglected. The power of the rotating disc $P = \tau \omega$, where $\tau$ is torque = 17.48 Nm and $\omega$ is angular velocity ($2\pi$ in 5 s). Work done/turn = 105 Ws, that is lighting a 21 W ($P_v$) bulb for 5 s.

**Figure 4:**
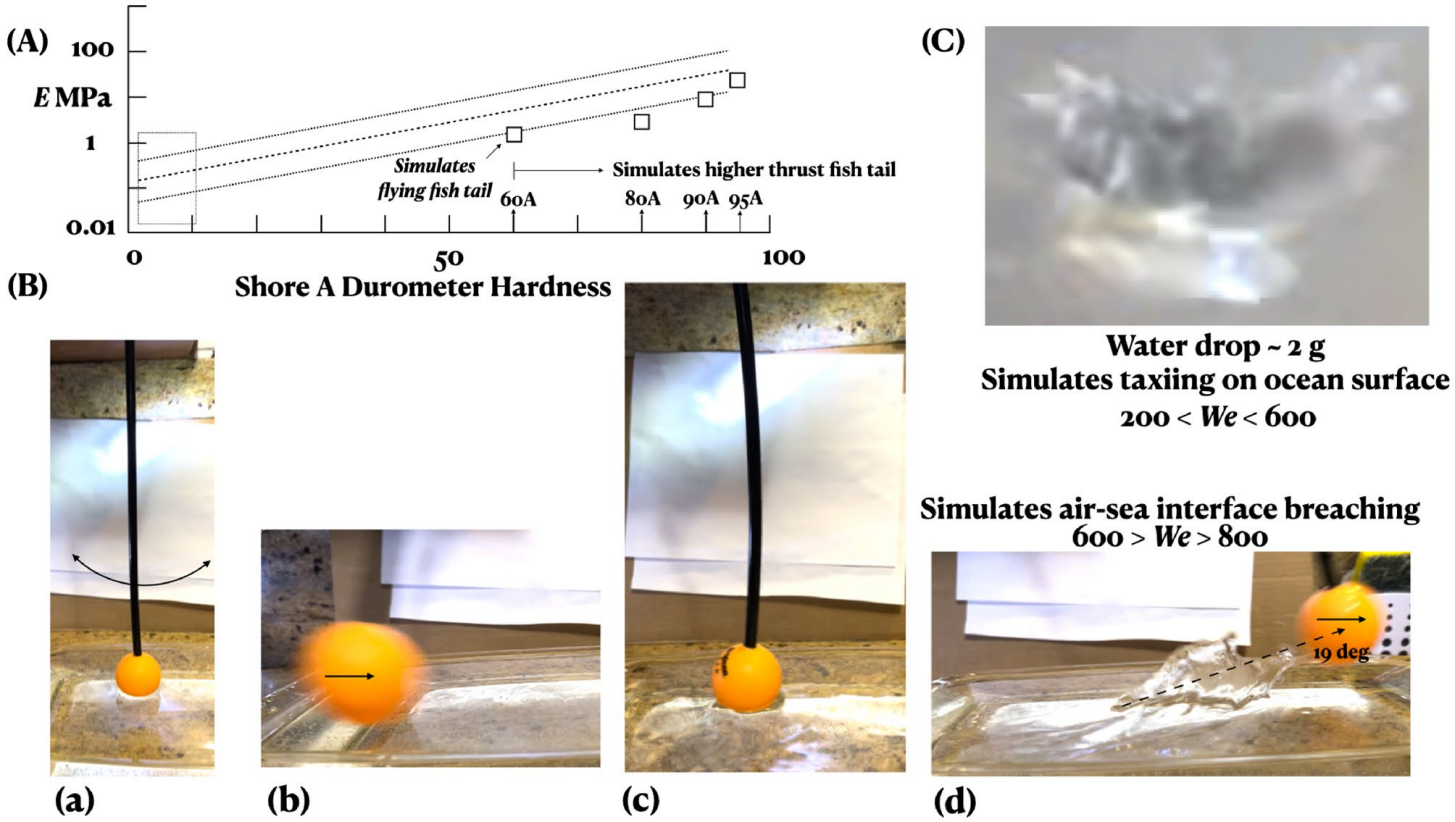
Low inertia shallow angle (19$^{\circ }$) interface breaching of escaping flying fish of low rigidity *EI*, simulated by a low hardness rod. A fish tail is a resonant oscillator of unique Strouhal number of 0.3 (= frequency of flapping x amplitude of flapping / cruising speed). (**A**) *E* of fish tail *EI* simulation; 6.35 mm diameter, 30.48 cm long, polyurethane black rod. Lines are Dow data:^59^ dashed (mean), dotted (2) and boxed (scatter band). Squares: this work. (**B**a–d Time sequence of experimental simulation of instability of air-sea interface breaching ("Methods" section);^1^ a yellow table tennis ball is attached to a 60A Shore hardness (**A**) rod. (d) Water surface strike angle of 19$^{\circ }$ and the crown and spikes reproduce the oceanic Weber number of flying fish interface breaching exactly. Rigidity *EI* of the flying fish tail is reproduced. (**C**) Simulation of ocean surface taxiing by flying fish when the tip of the triangular tail fin strikes the water surface and where* We* is lower than when emerging from underwater in (**B**). Given the low tail rigidity, a ballistic jump, which requires a high thrust, is beyond the capability and is not resorted to, indicating the low cost frictional nature of the last leg of the interaction. After all, the sailfish does not chase up to the free surface.

Does the flying fish school of 100 fish swimming at the diametrically opposed end and in synchrony accelerate the vortex generation? Assuming a steady solid body rotation, the upper limit of the total energy input is  52.5 Ws by the sailfish and  52.5 Ws by the flying fish school. An individual flying fish while circling is required to spend a maximum energy of 0.525 Ws. However, taking the cruising drag of a flying fish to be 0.03 N (Fig. 4, "Methods" section), at a cruising velocity of 0.10 ms$^{-1}$, in 5 s, the work done is 0.015 Ws. The energy contribution of the flying fish school for vortex churning is 1.5 Ws $<< 52.5$ Ws. Hence, the vortex churning energy expenditure by the flying fish school is negligible compared to the sailfish.

#### Mechanism of sound pressure generation

The constant phase ±70$^{\circ }$ wave pattern (Fig. 3), observed on the free surface, is modeled as the result of an acoustic event below where pressure waves are propagated from a monopole. When the vortex turning torque stops suddenly, the inertia forces and spatial variations become vanishingly small: $\nabla f$ = 0, a stationary (inflection) point of function *f* is created. The compressible vorticity $\omega$ equation reduces to the stationary form: $\partial \omega / \partial t = (1/\rho ^2) \nabla \rho \times \nabla p$, where the grads are vectors and $\nu$ is absent; vorticity is deposited at the inflection points^18,23,32,39^. The density interface distortions are amplified, a wavelength $\lambda$ is selected from the geometry and $\omega$ is deposited at the inflection points^39^.

Anchovies and shrimp bodies are acoustically reflective^40^. The sailfish-flying fish rotary motion consists of a diametrically opposed pair of concave mirrors, acting as poles, frozen at the instant that the radial waves are produced ($t = 0$). All poles lie on a circle of 5 m diameter. For 5 radial waves standing, $\lambda$ = 0.5 m. Speed of sound in water is 1440 ms$^{-1}$. The farthest radial wave from the center to where the sailfish is, was formed 0.0017 s ago = vortex breaking monopole formation time modeled earlier. It takes 0.0033 s for this wave to return from the flying fish, which is 0.005 s since when time $t = 0$ occurred.

The ray equation of the concave mirror is $1/f = 1/u + 1/v$, where *f*, *u* and *v* are radial distances of the focus, object and image from the pole. Because *f* is constant, *v* changes when *u* changes. Fig. 3a shows black circular arcs where the interfering waves are amplified. Because the monopole formation and first reverberation times are both 0.0018 s, the pure tone frequency of reverberation, 555 Hz is $\le$ 1 kHz, a human and fish audible range. Around 1 kHz, anchovy bladders amplify sound by a maximum of 8 dB when within the fish–a data applicable to flying fish and, when outside, up to 12 dB^40^.

Applying the man-made underwater explosion characteristic time of 1 ms (< 1.8 ms because of higher energy intensity), when the sailfish stops circling, in 1 ms the rotational energy of the vortex radiates from a monopole located at the center of the swimplane. Assuming vertical swimming, the flying fish bladder can adjust to a depth static pressure change of 0.10 ms$^{-1}$ while the sailfish can withstand 1 ms$^{-1}$, maybe even up to 7 ms$^{-1}$. So, while the sailfish has an ability to withstand a greater amplitude of static pressure change in 1 s, an energy release in 1 ms is far too quick for it. There is no evidence of bladder rupture. At the free surface, compared with the wind waves, the wave height initially is estimated at about 15 cm. So the shock is 1.4 kPa (0.2 psi) at the free surface before it starts decaying exponentially. In air, in a 5$^3$ m$^3$ room, a 20 W speaker is considered adequate which gives 115 dBm (ref. 1 mW) and 123 dBm if we add the maximum fish bladder amplification. This sound level is sufficiently disturbing but not high enough to burst the bladder.

The sailfish may complete $\pi$ turns to situate the school diametrically opposed, making the vortex power $P_v$ = 10.5 W. When the sailfish stops rotating, say $0.5P_v$ W is spent in pushing the water upward in the direction of minimum resistance, distorting the free surface against the gravitational force. Say $0.5P_v/3$ W is lost in the unconstrained nozzle circumference, and an equal amount of $0.5P_v/3$ W in the diffuser circumference, where there is no mirroring. The acoustic mirrors reflecting the incident shock waves cover 2x70$^{\circ }$, which is about 33% of the circumference of the swimplane and 2/3rd of the sailplane power is radiated to infinity. Therefore, power involved in mirroring is $0.2P_v$ W, that is 2.1 W. If 50% of $P_v$ is absorbed in the saltwater heating, then the effective shockwave and sound power is 1.0 W. The sound pressure level is then 90 dBm. With bladder amplification, the intensity is 98 dBm.

Compare the impact of the SPL range of 98 to 123 dBm on the physiology of fish and humans: there is a 50% risk of human fatality due to pulmonary or abdominal failure for 302±16 kPa-ms impulse^41^. For a square relationship of sphere area, the source pressure in the vortex 1 ms shock is 1.4 kPa $\times$ 5$^2$ = 35 kPa-ms which is 10% of the level for human fatality-a small but significant level.

The acoustic event estimates are: when $\lambda$ = 0.5 m, the maximum amplitude drops from $\approx$ 98−123 dBm to the background noise of $\approx$ $\le$ 70 dB in 5 s, the vibration frequency is $\le$ 1 kHz, the radial extent of the amplitude amplification due to the positive wave interference is  5 cm, the thickness of the black lines in Fig. 3a, which is equal to the size of the flying fish head, the diameter of the reverberation water chamber is 5 m, the sailfish focal point distance from the pole is 2.5 m, and the swimplane depth under the free surface is 2 m. The pressure impulse vibrates the bone that links the bladder to the ear which causes pain, as evidenced by gaping mouths^1^. The cilia polarization disorients the flying fish. The low energy cost effects of anthropogenic noise, found in Popper^36^, are likely to apply. The impulsive motion selects $\lambda$ = 0.5 m from the size of the vortex.

Due to the proximity of the flying fish to the noise source, the shock waves are spherical, applied uniformly on the body. The motion sequences are: the sailfish circles at 1 ms$^{-1}$; a 5 m diameter vortex is setup in the horizontal swimplane; the sailfish stops; the shocks/sound waves reverberate in the 5 m diameter ‘chamber,’ and then the school forms a tight ball with diameter $\le$ 2.5 m. When corralled to a packed ball, the flying fish try to swim to the center of the ball in order to shield from the ‘blast,’ which has already occurred. This event is marked as a $\lambda$ model in Fig. 3b. The surface wave pattern demonstrates the presence of $0.5 \lambda$ standing waves in the lower arc. Summed linearly, a ($\lambda + 0.5 \lambda$) model (Fig. 3b) results by locating the $0.5 \lambda$ flying fish reverberation origin at the singularity. The flying fish experiences two impulses, first, one instigated by the sailfish, and the other by the school itself.

Each flying fish produces a pair of dipoles due to tail flapping whose frequency *f* is given by the constant Strouhal number $St = fA/U$, where *A* is tail flapping amplitude and *U* is swim velocity. If *A* = 0.02 m, *U* = 0.1 m/s and *St* = 0.3, then *f* = 1.5 Hz. For the sailfish, if *A* = 0.1 m, *f* = 3 Hz. The interaction between the noise source and the still water mass is computed using the Euler–Lighthill (EL) equation: $(\partial ^2/\partial t^2 - c_o^2 \nabla ^2)\rho = (\partial ^2 T_{ij} / (\partial x_i \partial x_j)) (x, t)$, where $\rho$ is the density and $c_o$ is the sound speed in the medium. The Lighthill’s stress tensor is $T_{ij} = \rho u_i u_j + (p-c_o^2 \rho ) \delta _{ij} - \tau _{ij}$, where $u_i, p, \tau _{ij}$ are velocity, pressure and viscous stresses. Neglecting thermal effects, $T_{ij} = \rho u_i u_j$. The EL-equation has been solved numerically for a monopole noise source with and without the mean flow in a shear^16^. The numerical results, reproduced in Fig. 3c, compare remarkably well with the present model and the observed surface wave cluster. The weight of the evidence therefore confirms the acoustic nature of the predation. For adiabetic assumption, more fish in the school increase $T_{ij}$, increasing the noise impulse.

#### Vortex-stretching-induced intensification of SPL

The rotational kinetic energy of the conical vortex is given by $(\pi \rho r^2 h/3) (3r^2/20 + 3h^2/80) \omega ^2$, where $\omega = V_c/r$, $V_c$ is the sailfish cruise velocity, a constant. Here, *r*, *h* have large effects. To minimize cost, the sailfish swims inward making $r, h \rightarrow 0$ bringing the vortex closer to the free surface as it also tightens (recall $\partial p/ \partial r =\partial p / \partial z$), but not enough to entrain air because that would create costly waves^23^. When the sailfish *St* of tail flapping resonates with the laminar vortex core turning rate, the interaction creates inward spiraling bands undergoing stretching and vortex-vortex touching^42^. The conical vortex, stretched vertically to the maximum extent, bursts causing topological instability of the flying fish school, promoting vortex-vortex touching, breaking and monopole formation–all happening in about 2 ms.

To intensify $T_{ij}$, SPL and to compact the school, the conical vortex is axially stretched and the cochlear bands (in hurricane analogy) radially compact. The axial vorticity^23,43^ and the radial SPL receptivity as in the ’rain bands of hurricanes’, are intensified^44^. In the outer-inner layer interaction of a hurricane core, the circumferential Kelvin-Helmholtz vortex shedding (kh) is similar to fish tail flapping. The train of kh vortices is drawn inwards. Lateral translation of the vortex will not add to stretching. The long wing-tip aerodynamic vortices wander radially due to axial helical instability of the radial velocity that scales with 1 to 10 turnover time;^42,45,46^ but, the present vortex is too short for helical instability to be relevant.

Say the vortex core pressure is $P_c$ and the pressure outside the vortex is $P_o=\rho g h$. The pressure difference across the swimplane spiral is $\Delta p = P_o - P_c$. The equivalent sound pressure level (SPL) is $dB = 20 log (\Delta p / P_{ref})$, where $P_{ref}$ is the reference pressure for 0 dB, which is the threshold for human hearing, equal to 20 $\upmu$ Pa, or about 1.97$\times$e$^{-10}$ atmosphere. The vortex stretching mechanism of acoustic amplification in the inward spirals bears some resemblance to the mechanoelectrical amplification mechanisms found in the vertebrate cochlea^47^.

The vortex is similar to that in^23^. Say, the vertical shear is $\partial u/\partial y$, the vertical vortex axis stabilizes dynamically by stretching, which is given approximately by the term $\omega _x \omega _y \partial u/\partial y$ in the vorticity equation $D/Dt [(1/2)\omega _i \omega _i]=\omega _i \omega _j \partial u_i / \partial x_j + \omega _i \nu \nabla ^2 \omega _i$^43,48,49^. If the vortex is slightly tilted from the vertical, a vorticity intensification mechanism ensues. Vorticity intensification leads to increasing self-induction which tilts the axis further, as in a positive feedback loop, until the angle of principal strain of 45$^{\circ }$ is reached when any further increase leads to a spectacular bursting releasing elastic energy^23,43^. The relevance of vortex stretching-induced bursting to the sudden collapse of the fish school is appealing due to the low cost of the positive feedback loop.

Consider a rectilinear vortex stretched axially. The vortex stretching term, analogous to the current in a straight wire, is $\omega \nabla u$ and $\omega _x \omega _u \partial u/\partial y$ in a plane. The pressure-velocity turbulence diffusion per unit volume is $(-1/R^3)(p/\rho )u$, analogous to the magnetic field. Therefore, $(-1/R^3)(p/\rho )u$ $\propto$ $\omega _x \omega _y \partial u/\partial y$. This equation, analogous to electro-magnetism, supplements Lighthill’s stress tensor^16^. As the vortex is stretched, turbulence diffusion increases, that is, velocity fluctuations increase and pressure decreases-vortex core cavitation is possible. The vortex diameter decreases. Turbulence intensification directly increases acoustic radiation as given by the EL equation.

The stretching term destabilizes the vortex and the shear diffusion term stabilizes. The constant value of their ratio sets up the condition of vortex instability leading to oscillation and bursting when the vortex slant in the principal plane is perturbed. Once stretching reaches the maximum value at a slant of 45$^{\circ }$, the SPL level will also reach a maximum. The vortex will burst at these maximal conditions.

### Determination of the flying fish tail hardness via reproduction of air-sea interface instability

Figure 4 shows the experimental reproduction of the oceanic Weber number *We* of air-sea interface breaching by the escaping flying fish^19^. Say, the destabilizing inertia force is $F_u$ and the stabilizing capillary force is $F_\sigma$, the viscous force is $F_\nu$ and the gravity force is $F_g$. No surface wave is produced. Accounting for the surface tension, $We = F_u/F_\sigma = \rho u^2 d/\sigma$, where $\rho$ is the density, *u* is the velocity, *d* is a resistive length scale and $\sigma$ is the surface tension. Accounting for $F_\nu$, define Reynolds number $Re = F_u/F_\nu = ud/\nu$, where $\nu$ is the kinematic viscosity. Because the fish is partially submerged, consider Froude number $Fr = F_u/F_g = V/\sqrt{gh}$, where *V* is the wave velocity of the gravity wave and *h* is the depth of water. Accounting for the relative strength of the stabilizing forces, namely capillary and viscous forces, define Ohnesorge number $Oh = F_\nu / F_\sigma = \mu /\sqrt{\sigma \rho d} = \sqrt{We}/ Re$, which remains a constant for the same body because the air, seawater material properties do not change.

If *We* is critical^19^, then $F_u$ is critical because $F_\sigma$, which denotes air-sea material and fish dimensional properties, remains a constant. Similarly, because $F_\nu$ is also a material property, *Re* also must be critical. The converse argument is that *We* must be critical if *Re* is critical. Wake symmetry breaking takes place when *Re* rises to a critical value ($5 \le Re \le 25$) and in that condition, flapping thrust jumps^17^. Hence, *Oh* is also critical. For *Re* = 25 and $We=$ 600 to 800, $Oh =$ 1.0. That $Oh \le$ 1.0, means jetting is possible as indeed is found in the spike formations in Fig. 4. Ink jet technology works on this principle.

Define Froude number as $Fr=u/\sqrt{gL}$, where *u* is the relative velocity between the sea and the emerging flying fish and *L* is the length of the fish water line. To keep *Fr* low, the fish emerges at a shallow angle whence *L* is high and approaches the fish length. It is for this effect that the fish emerges at a shallow angle of 19$^{\circ }$ ("Methods" section). Due to the morning tropical breeze, the ocean surface may slip and the fish may emerge in the direction of the wind, lowering *u* and *Fr*. That emerging flying fish taxi and glide all in the same direction, supports the argument that the flying fish senses the wind direction while underwater from the toroidal subsurface rotation and aligns in the wind direction before emerging^1^. Hence, $Fr<$ 0.4, the last efficient speed. For $u=$ 0.10 ms$^{-1}$, $L=$ 0.15 m, half the fish length, $Fr =$ 0.12. The wavelength of the *Fr* surface waves formed $<< L$, that is, the waves are capillary waves ($\le$ 0.007 m wavelength), worst, at the boundary of capillary and gravity waves making the wave drag $\rightarrow 0$.

Consider the impulsive accelerating term $\nabla p$ on a water-air (suffix w, a) ($\rho _w>> \rho _a$) interface of $\nabla \rho$ under the gravitational acceleration *g* (Fig. 4). Receiving little resistance, water penetrates the air. As circulations $+\Gamma , -\Gamma$ deposit sequentially at the inflection points along the interface length, a single mode interface of wave number $k=2\pi /\lambda$ is formed. The single mode amplitude first grows linearly with time through symmetric crests and troughs. This mode is followed by the growth of multiple modes and nonlinearities when asymmetric crowns and spikes form. The tip of the spike rolls up into a crown. Small scale disturbances appear on the interface, developing into a chaotic regime^19,39^. In Fig. 4, there are nonuniformities in the spacing and the heights of the spikes meaning that extraneous perturbations contributing to nonlinearities are also growing. Hence, while the stabilizing forces remain the same, the destabilizing inertia forces are higher compared to when the most organized crowns and spikes first form at $We=$ 200^19^. The destabilizing force drops during taxiing after emergence, that is when the sailfish threat recedes ("Methods" section)^1^.

Fig. 4 is a time sequence of a medium soft (60A hardness) rod striking the water surface at 19$^{\circ }$^1^. The interface pattern shows $600> We > 800$^19^ and is similar to in the ocean ("Methods" section). That the emergence is at a shallow angle of 19$^{\circ }$ and a ballistic 90$^{\circ }$ exit is not undertaken for a faster escape means the thrust is 0.03 N and not 0.981 N for a 100 g flying fish (60A hardness and not 95A or 75D−Fig. 4A). Moreover, a taxiing (Fig. 4C) is not avoided for quicker gliding. The flying fish is not in a tearing hurry to escape−a surprise. But, then the sailfish does not chase the prey after the topology is fully bifurcated (Fig. 1b). The flying fish motion becomes even more friction limited swimming up breaching the interface at a shallow angle.

The *We* between the air-sea interface penetration and taxing ($600> We > 800$ in Fig. 4B vs. $200< We < 600$ in Fig. 4C) is definitely different (video time stamps in "Methods" section), which indicates the presence of multistability in the hydrodynamics, tail rigidiy EI and the olivo-cerebellar control of the flying fish tail oscillation^18^. The inertia force and disorganization are reduced while taxiing on the ocean surface than when emerging because the distance from the sailfish threat has increased. The multistability is not random, but chaotically controlled, depending on the threat perception.

## Discussion

The interaction–dispersion ($\theta _b, z$) bifurcation equation is similar to Turing’s reaction-diffusion equation. The effect of the tiny spatial in-homogeneity in the flying fish distribution at the bifurcation point ((0, 0) in Fig. 1b) can be modeled by coupled ($\theta _b, z$) equations^50^. The bifurcation will split into primary and secondary bifurcations similar to Fig. 1 in^50^. The result would model the emerging and gliding flying fish distributions seen in air above the compact patch of arced surface waves, thus connecting them back to the underwater bifurcation location. The surprising appearance and dispersion of a single easy-to-miss, fish-scale coherent wave patch on the free surface of the tropical ocean, in the midst of the wind and gravity waves, is a discovery being reported by this paper. It is expected that the correlation coefficient between the horizontal and vertical oscillations of the school, prior to bifurcation (Fig. 1b) increases and the bifurcation occurs when the coefficient exceeds a critical value. The tapered cloak boundary favors the upper branch to be leaner in escape population.

Oceanic Naval trawling shows biological resonant scattering by anchovy and shrimp schools. The fish swim bladders are of interest because they amplify sound. Taking the anchovy without the bladder as the baseline, sonic scattering studies show that the swim bladder alone amplifies 1.2 kHz sound by 18 dB while a live anchovy amplifies by 9 dB. In a 7.1 cm long goldfish, the amplification in the swim bladder alone is 12–14 dB at 0.5–2.5 kHz while the amplification is 6–7 dB in the fish. Bony fish, like flying fish, that use buoyancy have air bladders which are compressible and act as sound transformers. The neighboring bones amplify the sound. Particle motion is not the mechanism because the frequency is $>110$ Hz. The bones between the bladder and ears, the mechanical links, vibrate. The wave interference may cause a sudden bending of the polarized cilia in the fish ear, which are used for direction sensing, disorienting the flying fish^36^. Theoretically, the resonant frequency of a fish increases with depth. Models of reflection of resonant frequencies from fish show that for a given frequency, the target strength is greater for the side aspect than for the dorsal aspect. Further, the target strength increases with the size of the fish. That is, the ability of the sailfish in reflecting sound is higher than in an individual flying fish, but equals to the school. In shallow waters, the propagation loss due to fish populations is complex. The sailfish-flying fish interaction under consideration occurred in the early morning. It is unknown if the propagation loss increased or decreased when the acoustic predation occurred. However, in some populations there can be a drop during the early morning. The sailfish acoustic predation utilizes body concave mirroring, echo wave interference and precise spatial localization at the prey fish ear drums. The energy expense is lower than man-made noise. The dB level along the black lines in Fig. 3 may only be $>85$ dB as in humans threshold, but applied suddenly to startle (the bladder does not burst out of the mouth)^1^. The pile driving guideline of 150 dB re 1 $\upmu$Pa (rms) amplitude is irrelevant^41^. Underwater ambient SPL is as follows. In air, the corn popping mean SPL is 85 dBA^18,51^. In a controlled 200–300 Hz impulse of amplitude 2 psims for 1 ms in a 9.1 m deep tank the peak SPL is 185.5 dB (re 20 $\upmu$Pa) in-water, equivalent to 5.44 psi, causes no human hearing loss at 1006 m away^52^. The ambient SPL is $\le$ 70 dB, the quietest sea conditions at dawn. The ocean ambient SPL level near the free surface is $\approx$80 dB (Fig. 1)^18^ . In the UK, the ambient ocean noise is higher, $\ge$ the survey vessel. It is painful to humans when the intensity is $\ge$ 85 dB. The noise is unbearable at 120 dB (= disco noise; $\ge$ trawler noise)^15,51,53–55^. Because the noise is not prolonged, the high dB levels along the bold black lines in Fig. 3a is only what will intensify the SPL in the ears of the flying fish. For the same reason, the energy input in the present example of predation should be lower than more commonly studied man-made noise^13,15,36,55^. Masking is the hearing threshold above the near free surface oceanic noise which is 70 dB at dawn. Median ocean noise levels ranged in UK measurements from 81.5 to 95.5 dB re 1 $\upmu$Pa for 0.33 octave bands from 63 to 500 Hz^53^, but deeper in the ocean away from the UK shores, the noise level is closer to $\le$ 70 dB, also $\approx$ 70 dB re 1 $\upmu$Pa due to baleen whales, toothed whales, bottlenose dolphins and killer whales^55^.

An electrokinetic hypothesis of the mechanism of the putative fish pain, evidenced by the gaping mouths near topological bifurcation, can be tentatively proposed, where the fish scale acts like a nano-scale battery due to surface-normal ion flow, which is instigated by flow pressure gradient. The hypothesis draws analogy from graphene saline water nm scale batteries^56^. Putative fish pain is a controversial issue and new hypotheses may be useful. The electrocution is an explanation of why the sailfish captures only a few flying fish. The fish skin adsorbs saline water which is ionic^57^. Rainfall on land contains dissolved CO$^2$ from the surrounding air. The carbonic acid forms acidic rainwater which breaks rocks and the sodium Na$^+$ and chloride Cl$^-$ ions run into the ocean. The other less abundant ions in seawater are SO$_4^{2-}$, Mg$^{2+}$, Ca$^{2+}$, and K$^+$. Over time, the concentration of these ions increases. Commonly, assume that 35 g of salt is present in 1 kg of seawater. A vertical salinity variation of 5 mS is equivalent to 0.005 AV$^{-1}$. Coulomb and Faraday laws give an RC circuit where volts $\propto$ velocity. As per electrokinetic theory, an ionic solution under a pressure gradient produces electricity of energy  1 eV^56,58^. Due to electrochemical interaction, the fish skin will adsorb either a layer of Na$^+$ ions or Cl$^-$ ions-non-uniformly because the skin is nonuniform, or due to flow. Due to Coulomb force, an ion of the opposite sign is attracted to the first layer forming an electrical double layer. At the same time, a reverse Faraday’s electrolysis charge transfer takes place at the nm scale between the fish skin (scales) and the first ion layer creating a pseudo-capacitor which stores electrons with a potential difference along surface non-uniformities, or flow. The sailfish convects the ions containing water with gradients in the vertical, radial and circumferential directions along the streamlines. In the swimplane, the spiraling gradients accumulate the Na$^+$ and Cl$^-$ ions at where the flying fish congregate. The flying fish school experience a small induced current. The adsorptive flying fish skin and scales contain conductive saltwater and the nerves experience similar levels of 1 $\mu V$ perturbations^38^.

Compare sailfish with sail boat for verification of the model. Splitting of the sailfish’s sail into winglets and their reconstitution is comparable with the mainsail and jib sail management in sail boating during frequent tacking and gybing when the direction of a boat’s motion switches between port and starboard about the front or rearward wind direction by 45$^{\circ }$. Fig. 2f shows how the front and the rear winglets merge back into one sail. The boat-axial pitch between the mainsail and the jib increases between boat motion at 45$^{\circ }$, 90$^{\circ }$ and 135$^{\circ }$ with respect to the wind direction (close-haul to close reach, beam reach and broad reach). The converse, where the two split sails merge back into one, can be seen^1^ . In a sailboat, the sails loosen their camber in the ‘dead zone’ during tacking (‘luffing’) which de-powers the boat. Similarly, in the sailfish, a wave travels axially which oscillates the sail when the sailfish changes the sign of the camber. While the sailfish is self-propelled, the sailboat is not. The sailboat utilizes the resultant of the lift forces on the cambered sails produced by the incoming (external) wind and the reactive force imparted by the incompressible water on the keel and rudder of the boat. The thrust force reaches maximum value during the broad-reach (180$^{\circ }$) point of sail. The sail management has some similarity.

### Cloaking

The asymmetric bifurcation of the flying fish school (Fig. 1b), the formation of a monopole (Fig. 3), the reverberation between the concave acoustic mirrors (Fig. 3), are the principal features of the friction limited interaction (Fig. 4). These mechanisms remain sharply confined within a bell shaped vertical ‘cloak’ (Fig. 1a), otherwise acoustically isolated from the semi-infinite oceanic lowest dB noise ($p_{rms}$) space. The elementary mechanism of the cloaked control volume is the following. While *p* and $\rho$ are scalars, their grads are vectors. At the inflection points of the compression and rarefaction waves, the grads cross and minuscule vorticities are deposited, as per $\partial \omega /\partial t = (1/\rho ^2) \nabla \rho \times \nabla p$ , and these depositions are prone to nonlinear amplification^39^. The cloak therefore separates a relatively small conical baroclinic space where the grads of *p* and $\rho$ cross, depositing vorticity at the inflection points of the vorticity lines, in the midst of a vast barotropic surrounding space where the *p* and $\rho$ lines are parallel and do not cross. The cloaked region is hydrodynamically unstable, but the surrounding uncloaked region is not.

### Curiosity as the sailfish interaction motivation

Unlike for sardines, the sailfish herd does not drive up the flying fish school for easier feeding. Assume that, to the sailfish vision, the school of flying fish appears as a shimmering (blurred) blob in the horizontal plane with a ‘haunting’ monotonous, synchronized swishing of the tail flapping. Any individual fish may not be trackable unless it has a trajectory differentiated from the school. The sailfish being a larger animal is intelligent, and is motivated by curiosity, limiting the recruitment of inertia muscles and making the interaction friction based. The sailfish does not perceive the flying fish as a threat and yet, does not perceive it as too small to swallow either. The sailfish-flying fish inertia forces are proportional explaining why the flying fish also has the lowest thrust during interface emergence (Fig. 4) and a ballistic jump is not called for. This curiosity explanation tentatively explains the low capture rate of flying fish by the sailfish.

## Methods

### Sailfish vortex dimensions and moment and definition; see Fig. 1

The $70^\circ$ ($\approx 72^\circ$ ) sailfish arc is 3 m ($72^\circ \times 5 = 2\pi$) long. The vortex cone diameter in the sailfish swim plane is 5 m. The swim plane is located 2 m below the free surface. The estimated coefficient of moment is $100 C_M = 0.2$ at Reynolds number $Re = 3\times10^6$ (Fig. 1c). The ($C_M, Re$) estimate is in agreement with the one decade lower moment extrapolation of the laminar theory from all known laminar measurements. The estimate is also lower than all known measurements of moment at transitional and turbulent Reynolds numbers^24^. *The sailfish moment expended is negligible.* Define interaction = predator-prey separation $\Delta$ = $f(\theta _b,z)$, where $\theta _b = \Delta$ for $z=0$; *z* is vertical separation and $z = 0$ at $h = h_0$ (Fig. 1b).

### Time stamps^1^ where flying fish topological bifurcation takes place; see Figs. 1, 3, 4

The one-dimensional bifurcation of the equation $\theta _b z - z^3 = Dz$ in $\theta _b$ versus z coordinates takes place at time stamp 0:06 to 0:07 of 1:28 mt:s in BBC Life - Flying Fish^1^. The $19^\circ$ emergence of flying fish from subsurface and crown and spike air-sea interface instability are at 0:25 s (compare with Fig. 4). The surface wave cluster can be observed at 0:45 s to 0:51 s at the center of the left half of the frames.

### Winglet location vis-a-vis sailfish circulation distribution, see Fig. 2

The sail splits primarily into two winglets $W_1$ and $W_2$ roughly where the body divides into front and rear, where $\Gamma$ and span are the maximum and where the flow properties change. The front half has these properties. The axial pressure gradient is $\partial p/\partial x < 0$, that is, favorable and accelerating; the boundary-layer is laminar, thin and attached; the body axial curvature is concave in the pressure side, that is destabilizing and the suction side is convex and stabilizing; the axial gradient of the elliptic body cross sectional area *A* is $\partial A/\partial x > 0$, the boundary layer has thinning effect; $\partial \Gamma /\partial x > 0$; the streamlines near winglet-body junction are converging, that is, this is a line sink flow, if $s, \delta$ are the surface distance and the boundary layer thickness, $\partial \delta /\partial s < 0$. The rear half of the body and the sail has these opposed properties. The axial pressure gradient is $\partial p/\partial x > 0$, that is adverse and decelerating; the boundary-layer is laminar, thick and prone to separation; the body axial curvature is concave on the pressure side and destabilizing and convex on the suction side and stabilizing; the axial gradient of the elliptic body cross sectional area *A* is $\partial A/\partial x < 0$, the boat tail boundary-layer has thickening effect; $\partial \Gamma /\partial x < 0$; the streamlines near the winglet-body junction are diverging, that is, this is a line source flow and $\partial \delta /\partial s > 0$. Inflection in streamline is minimized. The streamlines follow the axial direction closely and not the spanwise direction. Circulation $\Gamma$ is load whose moment about the center of pressure determines the roll, pitch and yaw control force and moment laws. The circulation is front-loaded (Fig. 2c). The sail is multiply split in the ’boat tail’ where $\Gamma$ *is declining.*

### Polyurethane rod properties for simulation of flying fish tail hardness and drag; see Fig. 4

Polyurethane is tear-resistant, stronger than natural rubber and its measured density is 1.554$\times$10$^3$ kgm$^{-3}$, while the manufacturer’s D$^2$ value is 1.25$\times$10$^3$ kgm$^{-3}$ (manufactured by Polyurethane Corporation, Addison, IL, USA 60101), whereas the ocean water density is 10$^3$ to 1.06$\times$10$^3$ kgm$^{-3}$. Indicating that the flying fish tail hardness *EI*, where *E* is the Young’s modulus and *I* is the moment of inertia, is nearly reproduced, the rod bends marginally (Fig. 4). The hardness lowers in the sequence 75D, 95A, 90A, 80A to 60A, described as extra hard, hard, hard, medium hard, and between medium hard and medium soft. The 60A is the first hardness that bends marginally upon water surface strike. The 60A (*E* = 1.52 MPa) is slightly harder than pencil erasers. The *E* values compare well with the Dow Data^59^. The simulation rod diameter was 6.25 mm and the length was 0.3048 m . The 60A Shore hardness reproduced the interface spikes and crown the best compared to oceanic patterns^1^. Drag simulation (20−30 mN) was achieved by attaching the following to the end of the rod (Fig. 4): a table tennis ball of mass 3 g and 40 mm diameter; a water balloon of mass 2 g with a 1/4” steel nut inside. A 2 g water drop has $200> We > 600$, which reproduces the lower *We* of the flying fish tail strike on ocean surface during taxiing after emergence indicating multistability of *We*. The unstable *We* drops as the sailfish threat recedes.

## Data Availability

All data generated or analyzed during the modeling and experimental simulation study reported in the paper are available from the author on reasonable request.
